# Urinary TIMP-2 and IGFBP7 elevate early in critically ill postoperative patients that develop AKI

**DOI:** 10.1186/cc14367

**Published:** 2015-03-16

**Authors:** P Honore, LS Chauwla, A Bihorac, AD Shaw, J Shi, JA Kellum

**Affiliations:** 1 VUB, Brussels, Belgium; 2 VAMC, Washington, DC, USA; 3 UF, Gainesville, FL, USA; 4 VUSM, Nashville, TN, USA; 5 WB, Carlsbad, CA, USA; 6 PiU, Pittsburgh, PA, USA

## Introduction

Little is known about temporal changes in [TIMP2·IGFBP7] relative to injury in patients who develop AKI. In this analysis, we examined [TIMP-2·IGFBP7] in serial urine collections from the subset of Sapphire patients who were admitted to the ICU after major surgery.

## Methods

We stratified 238 Sapphire patients into three groups by their maximum AKI stage within 48 hours of the start of surgery using KDIGO criteria (No AKI, KDIGO 1, and KDIGO 2 to 3). Median TIMP-2·IGFBP7 values were calculated from all samples collected at 12 (±6)-hour intervals for 4 days following the start of surgery.

## Results

**Figure 1:**
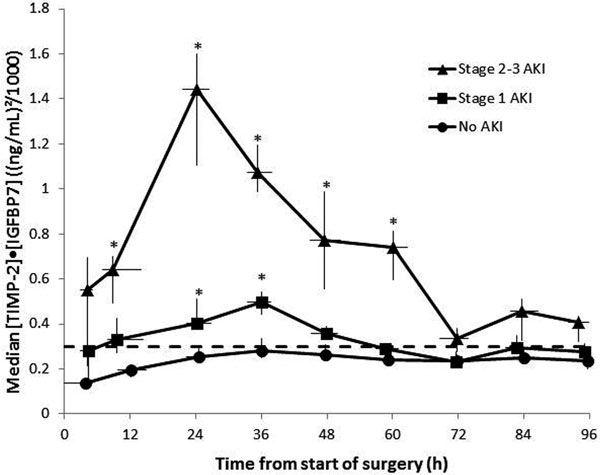


There were 101 patients without AKI, 95 patients with KDIGO 1 AKI, and 42 patients with KDIGO 2 to 3 AKI within 48 hours of the start of surgery. In patients without AKI, median TIMP-2·IGFBP7 values were less than 0.3 (ng/ml)^2^/1,000 (dashed line in Figure 1) for all time points. In patients with KDIGO 1 AKI, median [TIMP-2·IGFBP7] significantly exceeded this cutoff at 24 and 36 hours following the start of surgery (*one-sided *P *< 0.025). Median [TIMP-2·IGFBP7] increased earlier in KDIGO 2 to 3 AKI patients, remaining significantly elevated relative to the cutoff from 12 to 60 hours after the start of surgery. The highest median [TIMP-2·IGFBP7] was observed at 24 hours for KDIGO 2 to 3 AKI patients and was nearly five times the 0.3 (ng/ml)^2^/1,000 cutoff.

## Conclusion

Urinary [TIMP-2·IGFBP7] was significantly elevated as early as 12 to 24 hours from the start of surgery in patients who developed AKI within 48 hours. Monitoring of these biomarkers in the immediate postsurgical period might enable improved management of patients at risk for AKI.

